# The Activity of Native Vacuolar Proton-ATPase in an Oscillating Electric Field – Demystifying an Apparent Effect of Music on a Biomolecule

**DOI:** 10.3389/fmolb.2021.772167

**Published:** 2021-11-03

**Authors:** Pál Petrovszki, Krisztina Sebők-Nagy, Tibor Páli

**Affiliations:** Institute of Biophysics, Biological Research Centre, Eötvös Loránd Research Network, Szeged, Hungary

**Keywords:** H+-ATPase activity, biomembrane, oscillating electric field, sound stimulation of biomolecules, spectral characteristics of sound, rotary enzyme

## Abstract

The effect of an oscillating electric field generated from music on yeast vacuolar proton-ATPase (V-ATPase) activity in its native environment is reported. An oscillating electric field is generated by electrodes that are immersed into a dispersion of yeast vacuolar membrane vesicles natively hosting a high concentration of active V-ATPase. The substantial difference in the ATP hydrolysing activity of V-ATPase under the most stimulating and inhibiting music is unprecedented. Since the topic, i.e., an effect of music on biomolecules, is very attractive for non-scientific, esoteric mystification, we provide a rational explanation for the observed new phenomenon. Good correlation is found between changes in the specific activity of the enzyme and the combined intensity of certain frequency bands of the Fourier spectra of the music clips. Most prominent identified frequencies are harmonically related to each other and to the estimated rotation rate of the enzyme. These results lead to the conclusion that the oscillating electric field interferes with periodic trans-membrane charge motions in the working enzyme.

## Introduction

Beyond the obvious emotional effects, the psychological, physiological, and neurobiological effects of music on humans are well studied (Koelsch et al., 2016; Salimpoor et al., 2011; Xing et al., 2016; Ooishi et al., 2017). There is a long-standing interest also in the effect of music on non-conscious living matter. In most studies on organisms or biomolecules they are subjected to “listening” music from the air, and the observed effects are typically small and indirect (Wang et al., 2002; Zhao et al., 2002; Lestard et al., 2013; Oikkonen et al., 2016; Zhang et al., 2018). Our first objective is to search for a biochemical effect of a time-dependent physical quantity (other than pressure) derived from music. In a unique approach we convert music to alternating current and that to oscillating electric field (shortly AC field) and measure how the activity of an enzyme changes under the effect of that field. Yeast vacuolar proton-pumping adenosine-triphosphate (ATP) hydrolase (V-ATPase) is an optimal choice because it plays crucial roles in many life processes (Forgac, 2007; Vavassori and Mayer, 2014; Cotter et al., 2015; Sun-Wada and Wada, 2015) and it exhibits ATP rotary mechanism that involves periodic charge movements (Forgac, 2007; Ferencz et al., 2013; Cotter et al., 2015; Mazhab-Jafari et al., 2016; Ferencz et al., 2017).

It has been known for decades that the activity of enzymes embedded in membranes can be altered even by weak AC field because the field is amplified by the membrane, provided that the cells or vesicles are not leaky. An AC field in the order of 20 V/cm amplified by tightly sealed membrane vesicles can increase the activity of membrane-bound enzymes and even induce active transport by ATPases (Tsong and Astumian, 1986; Tsong et al., 1989; Liu et al., 1990). Such studies have hitherto most commonly utilised a sine waveform (Tsong and Astumian, 1986; Tsong et al., 1989; Liu et al., 1990; Holian et al., 1996). We have shown recently that V-ATPase is sensitive to AC field of pure sine waveform (Ferencz et al., 2017)*.* However, pure sine (or other regular waveform) oscillation hardly ever happens in living cells or in nature in general, and it has been suggested that it may not be the most efficient waveform in such studies (Treo and Felice, 2008). Therefore, our second objective is to test wether an AC field with a complex, non-regular time-dependence and a wide and variable frequency spectrum can affect enzymatic activity. Although musical electric oscillations are also not present in living cells (except most likely some cells related to hearing) it satisfies these requirements very well.

V-ATPase belongs to the family of membrane-attached biological macromolecules whose functioning involves a rotary mechanism (Stock et al., 2000; Forgac, 2007; Nakanishi-Matsui et al., 2010; Vavassori and Mayer, 2014; Cotter et al., 2015; Morales-Rios et al., 2015; Sun-Wada and Wada, 2015; Zhao et al., 2015). V-ATPase pumps protons across certain biomembranes and it is a key rotary enzyme in all eukaryotic cells, acidifying intracellular compartments and the extracellular space in some tissues (Nishi and Forgac, 2002; Forgac, 2007; Nakanishi-Matsui et al., 2010; Zhao et al., 2015). A class of proteins including its rotor proteins has functions independent from V-ATPase (Holzenburg et al., 1993; Arechaga et al., 2002; El Far and Seagar, 2011; Couoh-Cardel et al., 2016). V-ATPase is also a potential therapeutic target in several diseases (Farina and Gagliardi, 1999; Nishi and Forgac, 2002; Bowman et al., 2006; Forgac, 2007). V-ATPase works in the opposite sense as the better known F-ATP synthase (Sambongi et al., 1999; Stock et al., 1999; Stock et al., 2000; Bustamante et al., 2001; Yasuda et al., 2001; Nishio et al., 2002; Diez et al., 2004; Rondelez et al., 2005; Nakanishi-Matsui et al., 2010; Morales-Rios et al., 2015). F- and V-ATPases are true molecular engines (Stock et al., 1999; Grabe et al., 2000; Bustamante et al., 2001; Rondelez et al., 2005; Nakanishi-Matsui et al., 2010). In both enzymes, catalysis and proton transport are strongly coupled *via* the rotary mechanism (Farina and Gagliardi, 1999; Sambongi et al., 1999; Stock et al., 2000; Rondelez et al., 2005; Bowman et al., 2006; Forgac, 2007; Zhao et al., 2015).

**FIGURE 1:**
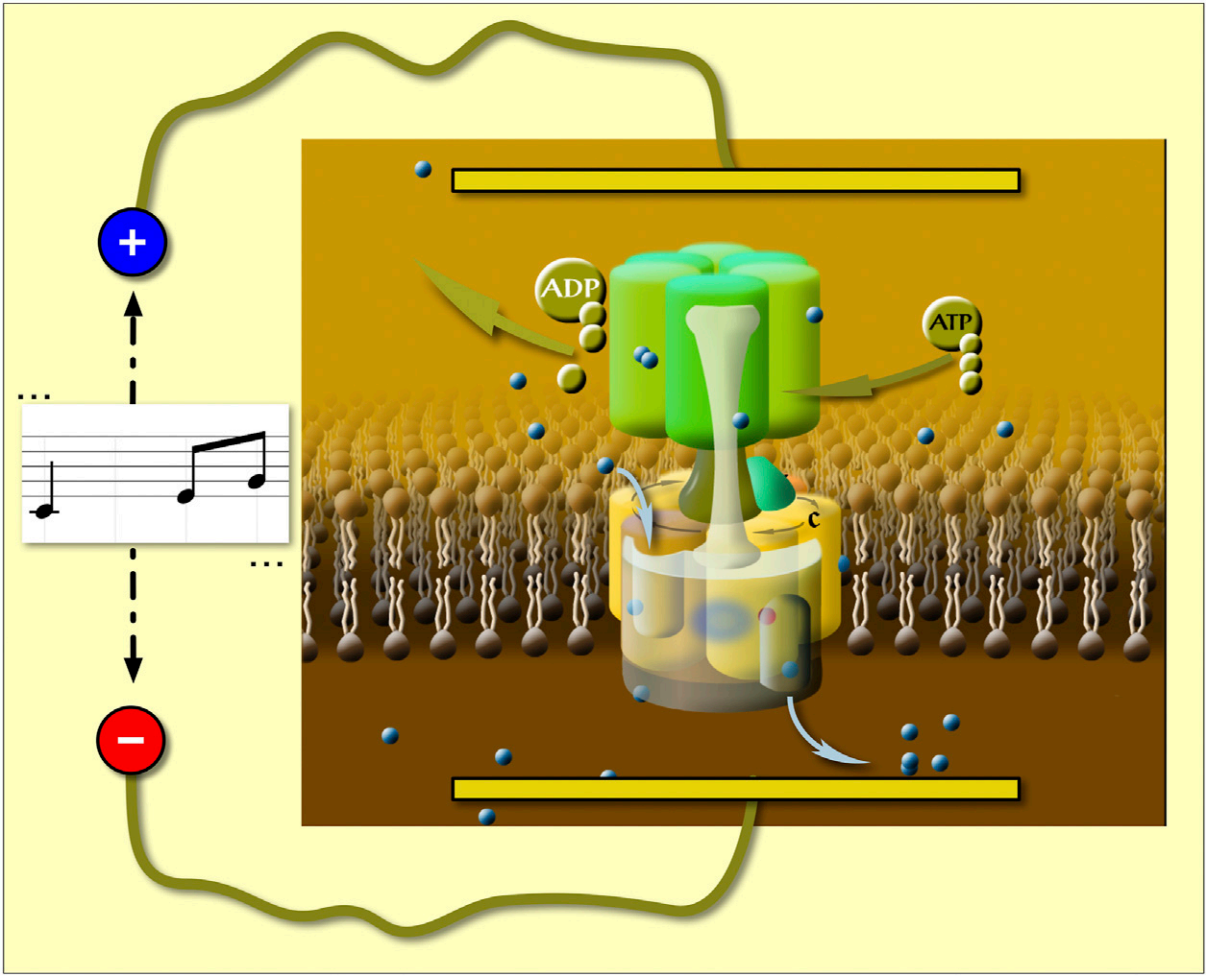
The oscillating trans-membrane electric field is parallel to the direction of the net vectorial proton transfer in V-ATPase [based on refs. (Ferencz et al., 2013; Ferencz et al., 2017)].

Since the periodicity of vectorial charge movements is related to that of the rotation, an oscillating electric field (EF) with the frequency of the main charge movements should have maximum effect on enzymatic activity. Indeed, in a first of its class measurement we applied AC field on a rotary enzyme (the V-ATPase) and discovered a new resonance-like frequency response of the enzyme to AC field, which allowed us to directly determine the rotation rate in native V-ATPase in its native membrane environment (Ferencz et al., 2017). In that experimental setup we used membrane vesicles with high concentration of native V-ATPase and two flat platinum electrodes were immersed in the vesicle suspension. We have proven that our vesicles are sealed and the membrane-amplified AC field acts on V-ATPase-related charge movements along the membrane normal (Ferencz et al., 2017). Under these conditions the EF sensed by membrane proteins (Figure 1) is amplified by 1.5 ^*^ cos(θ) ^*^ *R*_*0*_/*d* times relative to that in the aqueous phase (*R*_*0*_ and *d* are the radius of the vesicle and the thickness of the lipid bilayer, respectively, and *θ* is the angle between a line normal of the membrane surface with the EF, at the point of interest) (Tsong and Astumian, 1986). In the present study, the same experimental setup was used but with music in place of a sinusoidal waveform. Concanamycin A (ConcA) was used to determine the V-ATPase contribution to the total ATPase activity because it is a very specific and highly potent inhibitor of this enzyme. It binds to the intramembranous domain of V-ATPase and blocks rotation, hence proton transport and ATP hydrolysis (Farina and Gagliardi, 1999; Pali et al., 2004; Bowman et al., 2006).

## Materials and Methods

Yeast vacuolar membrane vesicles, natively hosting high concentrations of V-ATPase, were prepared as described earlier (Ferencz et al., 2013). The activity measurements with oscillating electric (AC) field applied on yeast vacuolar vesicles were also undertaken as described previously (Ferencz et al., 2017) except that here flat electrodes were positioned on the inner surface of a half-wide spectrometer cuvette of an optical path length of 1 cm. The assay mixture contained the activity buffer (50 mM MES/Tris, 5 mM MgCl_2_, pH 7.0) with a starting ATP concentration of 2 mM. ATP hydrolysis was thermostated at 20 ± 0.1°C for 10 min and then terminated. Phosphate liberated from ATP was then assayed photometrically (Ferencz et al., 2013). ATP was in excess, even at the end of the reaction period, so that the on-rate of ATP binding was not limiting the rate of ATP hydrolysis (Ferencz et al., 2013). Concanamycin A (ConcA) was used at a concentration of 1 µM. According to literature data (Muroi et al., 1993) and our study on ConcA inhibition of the yeast vacuolar V-ATPase (yielding a single ConcA binding site in the same vacuolar vesicle system) (Ferencz et al., 2013) 1 µM ConcA is sufficient to inhibit V-ATPase but it does not affect any other, e.g., secondary transport ATPases or P- or F-type ones (unlikely to be present in these samples anyway). V-ATPase constituted above 60% of the total ATPase activity in these vacuolar vesicles. The substrate was in excess (at 2 mM), and in these samples the difference in optical density (*OD*) is proportional to the concentration of ATP hydrolysed by V-ATPase in 10 min, that is to the specific V-ATPase activity (*SA*), as we have shown earlier (Ferencz et al., 2013; Ferencz et al., 2017). Under the same conditions as in this work, the V-ATPase activity peaks at 2 mM ATP, and that the time-dependence of ATP hydrolysis is linear over the 10 min reaction time (Ferencz et al., 2013).

**FIGURE 2:**
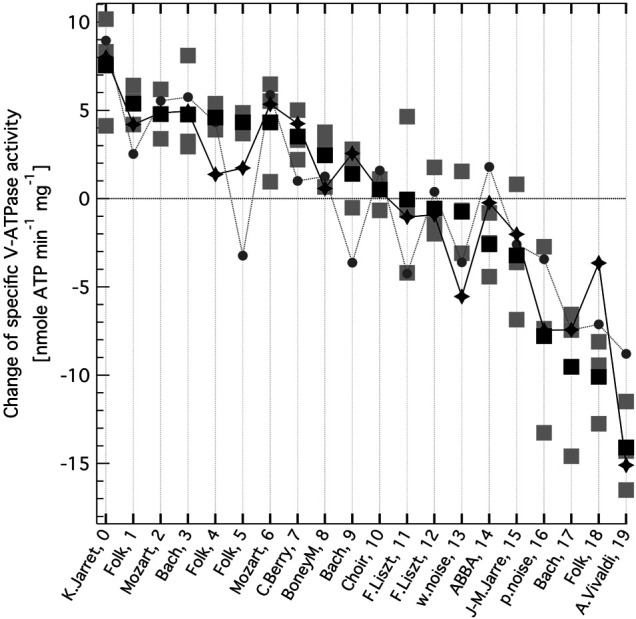
Change in the specific enzymatic activity of V-ATPase, as a consequence of exposing the enzyme to oscillating electric field generated from music. Changes are determined relative to the control, no AC field values. The zero line corresponds to the mean control (no AC-field) V-ATPase activity of 45.2 nmole ATP min^−1^ mg^−1^. The music sources from various genres and their audio file indexes are indicated on the x axis (see Supplementary Table S1 for more details). Grey squares are independent experiments, black squares are their mean values (All optical density (*OD*) and specific activity (*SA*) data are given in Supplementary Table S2) The summed intensity of the base frequency at 141.9 Hz and its first 4 overtones (grey circles) and that of 6 selected bands from an iterative search (black diamonds, see text) of the Fast Fourier Transform (FFT) spectra of the normalised music audio clips are also shown after best fitting linear scaling.

The AC field was applied with flat platinum electrodes (at a distance of 4.8 mm) in a sample cell with a cross section of 5.0 × 10 mm, under the same conditions as for the control. Delta (±ConcA) activity values were used as a measure of ATPase activity of V-ATPase in the presence and absence of the AC field, meaning four independent samples and a double subtraction for each experimental point in Figure 2. The experiments were replicated 3 times on the audio clips, which were taken from published CD tracks, summarised in Table 1 and described in detail in the Supplementary Table S1. The CD tracks were cut or restarted as required to get uniformly 5 min long audio clips. They were normalised in the digital domain to the same root-mean-square (rms) intensity of -22.53 dB. The clips were played from a Sony PCM-D50 digital recorder to a custom-built analogue amplifier, which was set such that 0 dB in the digital domain corresponded to 30 V peak voltage and, consequently, -22.53 dB corresponded to 2.24 V on the electrodes. The 30 V peak voltage yielded an effective peak electric field strength of 62.5 V/cm between the electrodes. The audio clips were played twice without interruption. Metric Halo (Safety Harbor, FL 34695, United States) hardware and software were used for real-time signal analysis and generation of the audio clips containing white and pink noise.

**TABLE 1:** The content of the audio clips^a^ played to the V-ATPase as oscillating electric field, sorted from maximum enhancement (top) to maximum reduction (bottom) of the specific activity of the enzyme.

Audio clip	Content
0_jarret	Jazz, piano: K. Jarret: The Köln concert, Part IIa (*D major*)
1_deszk	Folk songs for wine drinking, from West Hungary (*D major, A minor, A major; the pitch was not stable during the performance*)
2_mozart	W.A. Mozart: Divertimento D-dur, KV251, Rondeau, Allegro Assai (*D major*)
3_bach	J.S. Bach: Brandenburg Concertos, 5 in D-major, Allegro (*D major*)
4_deszk	Bagpipe-related folk songs from Nord Hungary (*A major >> A minor; bagpipe in the key of A is ∼ 20 cent flat*)
5_legedi	Hungarian Csángó folk song: Gergely dance (*G harmonic minor or C minor, solo instrument in key of C is ∼ 20 cent sharp, bass drum plays in C during almost the whole clip*)
6_mozart	W.A. Mozart: Eine kleine Nachtmusik, 1, Allegro (*G major*)
7_berry	C. Berry: Back in the United States (*Eb major, but in 430Hz tuning*)
8_boneym	Boney M: Megamix (*C major, C minor, E minor*)
9_bach	J.S. Bach: Toccata and Fugue in d-minor (*D minor*)
10_fchoir	Female choir: rearranged Hungarian folk songs (*G minor >> D minor > G mixolydian*)
11_liszt	F. Liszt: Buch der Lieder für Piano allein, No. 1 (*E major >> G major > F-sharp major*)
12_liszt	F. Liszt: Concerto for Piano & Orchestra in E flat major, 3, Andante (*E flat major*)
13_wnoise	white noise (not music)
14_abba	ABBA: Waterloo (*D major*)
15_jarre	Jean-Michel Jarre: Arpegiator (synthesizer music) (*C minor*)
16_pnoise	pink noise (not music)
17_bach	J.S. Bach: Brandenburg Concertos, 2, in F-major, Allegro (*F major*)
18_deszk	Wedding folk music from South Hungary (*A minor >> A major, C major*)
19_vivaldi	A. Vivaldi: The Four Seasons, Autumn, 1, Allegro (*F major*)

a Up to three of the most frequented key(s) and scale(s) are also given for each clip (referencing to 440 Hz tuning, unless indicated otherwise). Detailed source data of the audio clips are given in Supplementary Table S1.

Algorithms to perform numerical analysis in the frequency domain of the music audio clips were executed in Igor Pro (Wave Metrics, Lake Oswego, Oregon 97,035, United States) using built-in routines, e.g., Fast Fourier Transform (FFT), statistics or curve fitting, and custom code.

## Results

Table 1 lists the audio clips that were played to the platinum electrodes immersed in the aqueous suspension of yeast vacuolar vesicles with high concentration of active V-ATPase (Ferencz et al., 2013; Ferencz et al., 2017). A representative sampling of all music is impossible, the more so since we could not know what musical characteristics would be important, if any. Nevertheless, we had the following preferences: cover different dynamics and dominant pitch regions, include several genres and have different examples from some of the albums. We also included clips from white and pink noise. A detailed description of the source of each audio clip is given in the Supplementary Table S1. All audio clips were normalised to the same root-mean-square intensity. Double subtractions (released inorganic phosphate form ATP under AC field (in the absence minus presence of ConcA) minus the same in the absence of AC field) were undertaken for each set of experiments with each audio clip, yielding the change in the specific V-ATPase activity presented in Figure 2. The music clips are listed in Table 1 in the order from enhancement to reduction of V-ATPase activity. The original experimental *OD* and *SA* data are presented in Supplementary Table S2. Taking into account that subtraction increases relative error, the effect of music on V-ATPase is very clear and substantial. The mean difference of *OD* in the absence and presence of ConcA, without AC field (the control) was *OD*_*0*_ = 0.6126 ± 0.0042 (s.e.m.). The total protein per sample was set to 0.3 mg (Ferencz et al., 2013; Ferencz et al., 2017) and using our inorganic phosphate calibration on the same vacuolar vesicle system (Ferencz et al., 2013) the specific V-ATPase activity is *SA*_*0*_ = ∼ 45.2 nmole min^−1^ mg^−1^ in the absence of any AC field, corresponding to the zero line in Figure 2. The difference in specific V-ATPase activity between the most stimulating and most inhibiting music is more than ∼ 10 times the experimental error and amounts to ∼ 50% of V-ATPase activity under no AC field conditions. Some of the music clips are stimulating whereas others are inhibiting the enzyme, as opposed to the sine waveform, which was inhibitory over the whole audio frequency range except for a narrow region (Ferencz et al., 2017).

**FIGURE 3:**
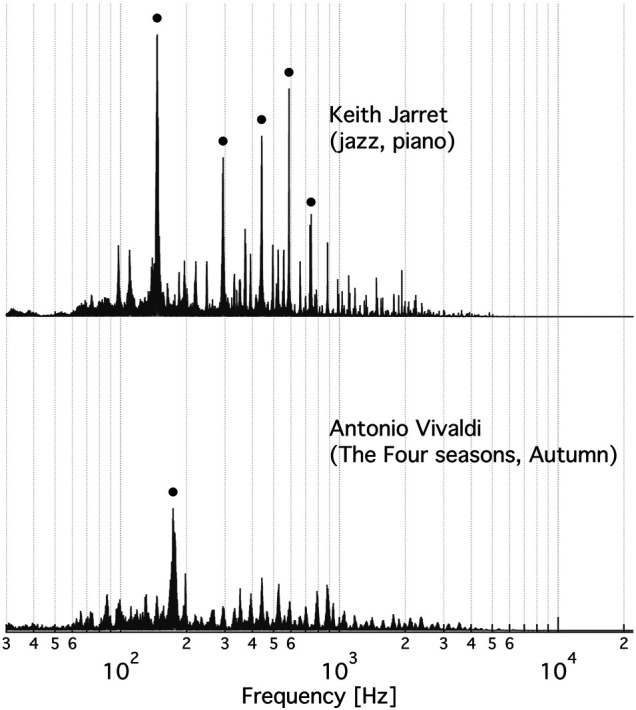
The FFT spectrum of the music clip that most stimulates **(top)** and most inhibits **(bottom)** the specific activity of V-ATPase. Top: An excerpt from Keith Jarret’s Köln concert (jazz, piano). The black circles indicate the five highest peaks at 146.7 ± 1.9 Hz, 293.4 ± 3.3 Hz, 440.9 ± 2.9 Hz, 587.9 ± 1.9 Hz, and 742.5 ± 1.2 Hz. Bottom: An excerpt from the Autumn of Antonio Vivaldi’s Four Seasons. The black circle marks the peak at 174.9 ± 5.3 Hz. The ± frequency intervals indicate the line-width of a Gaussian function fitted to the respective peak.

We found not any audible characteristics of the music (or musical genres, performers or composers) that could account for the preference order of V-ATPase. However, in the music most stimulating V-ATPase a single note dominates during the 5 min clip. That musical note is ∼ D3 (in 440 Hz tuning) at a frequency of ∼ 147 Hz. Consequently, this frequency not only gives the highest peak in the Fast Fourier Transform (FFT) spectrum of this clip but the four next highest peaks are its first four overtones (within the tolerance of the bandwidths) (Figure 3, top). The spectrum of the most inhibitory clip (Figure 3, bottom) shows a dominant peak at ∼ 173 Hz, which corresponds to a frequented musical note in that piece, F3 (in 440 Hz tuning), and there are no strong overtones present in the spectrum. These observations suggest that the ∼ 173 Hz band is inhibiting and the ∼ 147 Hz band, and possibly some of its overtones, are stimulating V-ATPase, respectively. Our previous calibration (Ferencz et al., 2013) can be used to estimate the mean rate of the 60-degree rotation steps of V-ATPase (Ferencz et al., 2017) in the absence of AC field, using the above no-AC *OD* value: it is ∼ 59 Hz in the absence of AC field (corresponding to the zero line on Figure 2) and ∼69 Hz under the most stimulating music clip. Interestingly, the above D3 note is reasonably close to the first overtone of this rotation frequency. The above observations prompted us to check the musical keys and scales of the clips, which are also indicated in Table 1 (and in the Supplementary Table S1 with more details). Astonishingly, the top four clips stimulating V-ATPase are exclusively or, in one case, in part in the musical key of D, and the next three clips are in the harmonically closest keys of A and G. The bottom end of the list is less consistent in this respect, but the five most inhibitory clips include the pink noise, two clips are in the key of F and one clip is in the harmonically closest key C. However, these musical qualifiers alone can not explain the sign and magnitude of the AC effect.

**FIGURE 4:**
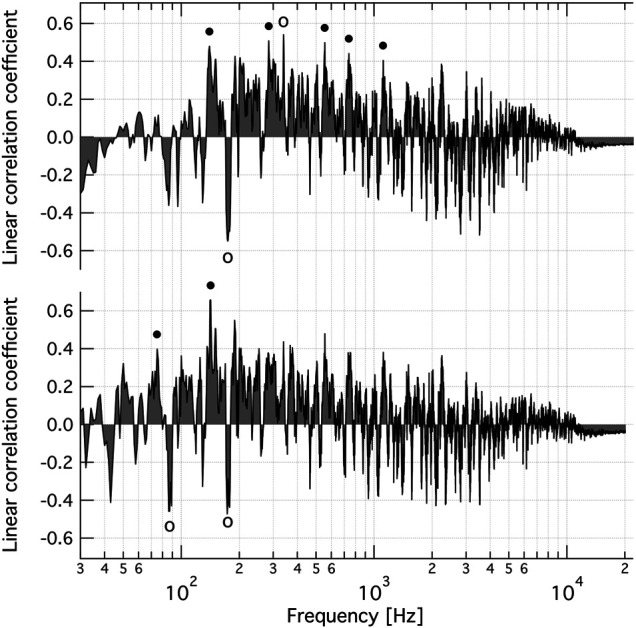
Frequency dependence of the coefficient of linear correlation between band intensity of the FFT spectra of normalised music clips and changes in specific V-ATPase activity caused by oscillating AC field of musical content, after best fitting linear scaling. Top: Circles indicate harmonically related peaks at 146 ± 12, 285.8 ± 8.0, 559 ± 21, 743 ± 31, 1,127 ± 43 Hz (solid circles) and 175.4 ± 4.9, 338.9 ± 4.8 Hz (open circles). The bandwidth was 1.4 Hz. Bottom: Base frequency plus the first 3 overtones. Circles indicate the harmonically related peaks at 73.4 ± 5.2 and 143 ± 15 Hz (solid circles) and 87.1 ± 2.3 and 175.0 ± 4.2 Hz (open circles). The bandwidth was 0.22 Hz. The ± frequency intervals indicate the line-width of a Gaussian function fitted to the respective peak.

We have done extensive time-domain analysis of the music audio clips (not shown) but that provided no insights vis-à-vis the “preference list” of V-ATPase. Therefore, we tested to discern whether single frequency bands correlate with the experimental effect. This was accomplished by computing the summed intensity *I(f)* of a band centred around frequency *f* for each audio clip, and the series of 20 intensities was linearly fitted *via* least squares to the experimental data to minimise *∑*(*∆SA*_*i*_ *-* (*a + b* ^*^ *I*_*i*_ *(f)*)^*2*^, where *i* indexes the independent experiments, and *a* and *b* denote fitting parameters. The linear correlation coefficient between the fitted intensities and experimental data was then calculated. It should be noted that if the best fitting *b* parameter is negative, the corresponding frequency band correlates negatively with experimental data, i.e., more of that frequency in the spectrum leads to a stronger inhibitory effect on the enzyme. Figure 4 (top) shows the linear correlation coefficient versus the frequency of a single frequency band. The effect of the bandwidth was also tested and Figure 4 (top) was constructed with a fixed bandwidth of 1.4 Hz yielding the highest global maximum. Four of the six prominent positive peaks are harmonically related to (are integer multiples of) a base frequency at 146.7 ± 1.9 Hz, whereas the global maximum at 338.9 ± 4.8 Hz is close to the first overtone of the frequency, 175.4 ± 4.9 Hz at the global minimum. (The ± frequency intervals indicate the line-width of a Gaussian function fitted to the respective peak.) These latter observations suggest that, in addition to the weights, overtone phase is also important.

Whether specific harmonic structures yield a better linear correlation was then tested. Figure 4 (bottom) shows the linear correlation coefficient between the experimental *∆SA*_*i*_ values and the least-squares fitted combined spectral intensity of a base frequency plus its first three overtones as a function of the base frequency. The weight of the partials (*w*_*j*_, *j* = 0,1,2,3) were set to decrease proportionally such as *w*_*j*_ = *w*_*0*_ * *f*_*p*_^*j*^, and the decay factor (0 < *f*_*p*_ < 1) was also a fitting parameter with a best fitting value of 0.641 ± 0.086 (fitting error, f.e.). The best fitting bandwidth was 0.223 ± 0.088 (f.e.) Hz. The base frequency yielding the highest (positive) linear correlation with the experimental data is again the 143 ± 15 Hz band, and the largest negative correlation is again at 175.0 ± 4.2 Hz. The first subharmonic peaks at 73.4 ± 5.2 and 87.1 ± 2.3 Hz are also reasonably prominent. Allowing for only even or odd overtones led to weaker correlation. If the first four overtones (as suggested by the single frequency plot, Figure 3, top) are used and if the individual weights, relative to the base, of the overtone band are also fitting parameters then the linear correlation coefficient is 0.776, with a base frequency of 141.9 Hz and weights (*w*_*j*_, *j* = 0,1,2,3) of 0.48 ± 0.53, 1.06 ± 0.70, 1.09 ± 0.60, and -2.4 ± 1.3 (f.e.), for the 1st, 2nd, 3rd, and 4th overtone, respectively, and with a bandwidth of 0.210 ± 0.050 (f.e.) Hz. The corresponding data are shown in Figure 2 (grey circles).

Finally, an iterative search was performed without enforcing any overtone structure and with released relative weights of the frequency bands, as follows. Frequency #1 was fixed at 338.9 Hz (starting with ∼146 Hz instead did not make any difference) and a plot was constructed of the linear correlation coefficient (between the *∆SA*_*i*_ data and the summed intensity of two bands) with a best fitting weight of a second frequency band where the independent variable was the frequency of that band. The position of the global maximum of the plot was spotted as frequency #2. Next, frequencies #1 and #2 were fixed, and a combined intensity plot was constructed as a function of frequency #3. This procedure was repeated up to frequency #6 (there was no significant improvement above 6 bands). Then a common bandwidth was optimised and all the weights and frequency values were adjusted in a fully released fit. A linear correlation coefficient of 0.861 was obtained, the highest so far, with frequencies #1-6 of 137.12 ± 0.22, 339.82 ± 0.60, 1,342.2 ± 1.6, 472.3 ± 0.25, 198.9 ± 0.10, and 174.69 ± 0.18 Hz and with corresponding weights of 1.03 ± 0.38, 1, 0.57 ± 0.17, 0.55 ± 0.23, 0.19 ± 0.21, and -0.64 ± 0.18 (f.e.), and a bandwidth of 1.09 ± 0.12 (f.e.) Hz. (These scaled intensities are shown in Figure 2, black diamonds.) Some of these frequencies are close to the harmonically related ones identified above. The ∼ 199 Hz band is the least significant, with the smallest weight and a large fitting error. The ∼ 175 Hz band has a negative weight, in agreement with the inhibitory character already identified for this band. However, new frequencies also appear in the list, suggesting that the charge movements in the rotary cycle of V-ATPase are complex and the effect of the AC field of musical origin on the specific V-ATPase activity cannot be fully described with just a few harmonically related frequency bands.

## Discussion

It has been hypothesised long ago that membrane ATPases could be affected or even regulated by oscillations of the membrane potential even in living cells depending on the frequency spectrum of those oscillations (Astumian, 1993). An AC field generated from music represents oscillations with a rather wide and dynamic frequency spectrum. Since V-ATPase showed great sensitivity to an AC field of the sine waveform (Ferencz et al., 2017), it was demanding to test wether it can react differentially to AC field generated from various music of different spectral characteristics. The applied peak EF strength of 62.5 V/cm may seem to be too small for an effect, however the sealed vesicle membrane amplifies the EF for membrane proteins, according to fundamental studies showing that even 20 V/cm external AC field induced active transport by Na,K-ATPase (Liu et al., 1990; Tsong and Astumian, 1986; Tsong et al., 1989). In our case, the amplification factor is ∼ 1.5^*^300nm/5 nm = 90, considering the dimensions of the membrane vesicles (Ferencz et al., 2013; Tsong and Astumian, 1986). As opposed to living cells, in our experiments all vesicles and V-ATPase molecules are exposed to the same external field, maximising the effect. Indeed, even a weaker external AC field, with a sine waveform, was effective on V-ATPase in the same vesicle preparations (Ferencz et al., 2017). In our samples V-ATPase sits in its native membrane environment but a static trans-membrane potential is not present in these vesicles. On the other hand, although a stationary transmembrane potential is present in living cells, it has been suggested that only a “locally” oscillatory or fluctuating EF can be used by an enzyme to drive a chemical reaction away from equilibrium (Tsong et al., 1989).

Due to the angular dependence of the membrane-amplified EF (Tsong and Astumian, 1986) V-ATPases (distributed homogeneously in the vesicle membrane) sense different AC field strength, with a maximum and no effect in the two poles and the equator regions, respectively, with respect to the direction defined by the electrodes. In addition, the momentary inside-outside orientation of the AC field is always opposite in the two hemispheres of a vesicle, relative to the EF axis. Therefore, the EF is in the same and opposite direction of proton transport (which is always from outside to inside the vesicle) for 50% of the V-ATPases in each direction. Earlier we provided a model for how the AC field (of a sine waveform) can either inhibit or stimulate ATP hydrolysis by V-ATPase depending on the frequency of the oscillation (Ferencz et al., 2017). The fact that we have observed the theoretical maximum reduction (50%) of ATPase activity at very low AC frequency (Ferencz et al., 2017) implies that most V-ATPases are affected in our preparations.

We found no evidence that V-ATPase would have any musical preferences with respect to genres or performers. However, the enzyme shows substantial sensitivity to certain spectral characteristics of the AC signals. V-ATPase is driven by ATP hydrolysis but the AC field can affect enzymatic activity. The identified frequencies are in a perfect agreement with the “stimulatory and inhibitory musical keys”, despite the fact that the musical key and scale do not alone determine the spectrum. The intensity of certain frequencies, including overtones of the estimated rate of the 60-degree rotary steps of V-ATPase, exhibit a convincingly high correlation with the effect of the AC field of musical origin on ATPase activity. This suggests that well designed specific waveforms must be more efficient in affecting V-ATPase than the pure sinusoidal waveform, which is the most widely used waveform in studies of the effect of EF on biomolecules (Tsong et al., 1989; Holian et al., 1996; Treo and Felice, 2008). This conclusion is in agreement with previous suggestions (Tsong and Astumian, 1986; Liu et al., 1990; Treo and Felice, 2008) and needs further studies, preferably in a real-time enzyme activity monitoring setup. Although we found a very good correlation between the activity changes and band intensities of a composition of certain frequencies of the music clips, we encourage other interested researchers to pursue research in this domain (we are willing to provide all data and assistance) to determine whether other temporal or spectral parameters yield even better correlation with the observed “musical preferences” of V-ATPase and how the new phenomenon depends on the physical conditions.

Finally, we strongly advise against any generalisation of our reported data (in particular Table 1) or using them in a non-scientific context. Firstly, since the activity of any membrane transporter depends on many conditions (such as, e.g., enzyme and substrate concentration, pH, temperature, lipid environment, hydration level, etc.) our observed specific activities are valid only under the present well-controlled conditions. Different conditions would yield different specific activity, hence different mean rotation rate, prominent frequencies, and different stimulation-inhibition order of a set of music clips. Secondly, the signals never took the form of acoustic music (pressure waves) in the present experiments, since the digital representation of music (recordings using microphones, amplifiers and analogue-digital converters) was used to create an oscillating voltage on the electrodes immersed into the samples. This is a different biophysical process from exposing living organisms to pressure waves. Thirdly, although pressure (and mechanical) waves can be converted to oscillating trans-membrane potential in organisms (this is actually happening in hearing organs), we are not aware of any report in which acoustic waves (such as music) generate a macroscopic electric field in a biomolecular system, which is achieved in this study (without acoustics) using an experimental setup with electrodes. The potential effect of (non-macroscopic) local oscillating trans-membrane potential (originating from, e.g., acoustic music) on membrane-bound enzymes demands further pioneering research.

## Data Availability

The original contributions presented in the study are included in the article/Supplementary Material, further inquiries can be directed to the corresponding author.
