# Supplementary material for: The Activity of Native Vacuolar Proton-ATPase in an Oscillating Electric Field – Demystifying an Apparent Effect of Music on a Biomolecule
Source: Front Mol Biosci. 2021 Nov 3;8:772167. doi: 10.3389/fmolb.2021.772167 (PMC8595334; doi:10.3389/fmolb.2021.772167)
Supplement: Supplementary file 1 [file Table1.pdf]

Supplementary Table S1. The content and source of music audio clips used in this paper, sorted from enhancement to inhibition of vacuolar proton-ATPase*

Audio clip	Content	Source
0_jarret	K. Jarret: The Köln concert, Part IIa (jazz, piano)	CD: Keith Jarret: The Köln Concert. Track #2. Performer: Keith Jarret, piano. Publisher: ECM records, München, Germany, 1975 (CD id 1064/65). (*D major, D note dominates the clip*)
1_deszk	Folk songs for wine drinking, from West Hungary	CD: Mögüzenöm a rózsámnak. Track #11: Borivó nóták (Dunántúl). Performer and Publisher: Deszki Népdalkör, Deszk, Hungary, 2010 (non-commercial release). (**D major, A minor, A major**; *the pitch was not stable during the performance*)
2_mozart	W.A. Mozart: Divertimento D-dur, KV251, Rondeau, Allegro Assai	CD: Music Digital Collection – 45, Mozart. Track #13. Performer: Camerata Academica Salzburg, Sandor Vegh. Publisher: GEMA, Berlin, Germany (CD id B 648). (**D major**)
3_bach	J.S. Bach: Brandenburg Concertos, 5 in D-major, Allegro	CD: Zyx Classic: J.S. Bach: Brandenburgische Konzerte. Track #13: 5 in D-major, Allegro. Performer: Südwest-Studioorchester, Dir. Heribert Münchner. Publisher: Bernhard Mikulski Schallplatten-Vertriebs-GmbH, Elbtal-Dorchheim, Germany (CD id CLS 4032). (**D major**)
4_deszk	Bagpipe-related folk songs from North Hungary	CD: Mögüzenöm a rózsámnak. Track #12: Felvidéki dudanóták. Performer and Publisher: Deszki Népdalkör, Deszk, Hungary, 2010 (non-commercial release). (**A major >> A minor**; *bagpipe in the key of A is ~20 cent flat*)
5_legedi	Hungarian Csángó folk song: Gergely dance	CD: Legedi László István: Csobános. Track #3: Gergelytánc. Publisher: Dialekton Népzenei Kiadó, Budapest, Hungary, 2006 (CD id BS-CD 05). (*G harmonic minor or C minor, solo instrument in key of C is ~20 cent sharp, bass drum plays in C during almost the whole clip*)
6_mozart	W.A. Mozart: Eine kleine Nachtmusik, 1, Allegro	CD: Music Digital Collection – 45, Mozart. Track #1. Serenade No. 13 G-dur KV 525. Performer: Franz-Liszt-Kammerorchester, János Rolla. Publisher: Music GEMA, Berlin, Germany (CD id B 648). (**G major**)
7_berry	C. Berry: Back in the USA	CD: Chuck Berry. Track #10 (the first 2.5 minutes is repeated). Performer: Chuck Berry. Publisher: Signal, Israel, (CD id 50650). (*Eb major, but in 430Hz tuning*)
8_boneym	Boney M: Megamix	CD: The greatest hits, Boney M. Track #14: Boney M Megamix: Rivers of Babylon, Sunny, Daddy Cool, Rasputin (the first 2.5 minutes is repeated). Performer: Boney M. Publisher: Telstar Records, Plc., 1992 (CD id TCD 2656). (*C major, C minor, E minor*)
9_bach	J.S. Bach: Toccata and Fugue in d-minor	CD: Die Grossen Meister der Klassischen Musik. Track #8. Performer: Hannes Kästner. Publisher: Delta Music GmbH, Königsdorf, Germany, 1993 (CD id. 11501). (**D minor**)
10_fchoir	Female choir: rearranged Hungarian folk songs	Concert CD. Track #16: Karai J.: Estéli nótázás. Performer and Publisher: Szeghy Endre Pedagógus Női Kar, Szeged, Hungary, 2012 (non-commercial release). (**G minor >> D minor > G mixolydian**)
11_liszt	F. Liszt: Buch der Lieder für Piano allein, No. 1	CD: Liszt Ferenc, Piano Concerto, Opus Postumum. Track #6. Performer: Jenő Jandó, Hungarian State Orchestra, Lamberto Gardelli. Publisher: Hungaroton, Budapest, Hungary, 1991 (CD id HCD 31396). (*E major >> G major > F-sharp major*)

12_liszt	F. Liszt: Concerto for Piano & Orchestra in E flat major, 3, Andante	CD: Liszt Ferenc, Piano Concerto, Opus Postumum. Track #3. Performer: Jenő Jandó, Hungarian State Orchestra, Lamberto Gardelli. Publisher: Hungaroton, Budapest, Hungary, 1991 (CD id HCD 31396). (**E flat major**)
13_wnoise	White noise (not music)	Generated with software.
14_abba	ABBA: Waterloo	CD: The ABBA Remasters, ABBA. Track #12 (the first 2.5 minutes is repeated). Performer: ABBA. Publisher: Polar Music International B.V., PolyGram, 1997 (CD id 533983-2). (*D major*)
15_jarre	Jean-Michel Jarre: Arpegiator (synthesiser music)	CD: Jean-Michel Jarre, Musik aus Zeit und Raum. Track #5. Performer: Jean-Michel Jarre. Publisher: PolyGram, Hanover, Germany (CD id 815686-2). (*C minor*)
16_pnoise	Pink noise (not music)	Generated with software.
17_bach	J.S. Bach: Brandenburg Concertos, 2, in F-major, Allegro	CD: Zyx Classic, J.S. Bach: Brandenburgische Konzerte. Track #5. Performer: Südwest-Studioorchester, Dir. Heribert Münchner. Publisher: Bernhard Mikulski Schallplatten-Vertriebs-GmbH, Elbtal-Dorcheim, Germany (CD id CLS 4032). (**F major**)
18_deszk	Wedding folk music from South Hungary	CD: Mögüzenöm a rózsámnak. Track #4: Délvidéki lakodalmas (Alföld). Performer and Publisher: Deszki Népdalkör, Deszk, Hungary, 2010 (non-commercial release). (**A minor > C major, A major**)
19_vivaldi	A. Vivaldi: The Four Seasons, Autumn, 1, Allegro	CD: Music Digital Collection – 15, Vivaldi. Track #7: Concerto No. 3 F-major, RV 293, ”Autumn”, 1, Allegro. Performer: Budapest Strings, Károly Botvay. Publisher: GEMA, Berlin, Germany (CD id B 518). (**F major**)

*The most frequented key(s) and scale(s) of the clips are given (referencing to 440Hz tuning, except when indicated otherwise) in boldface for those tracks for which they were indicated on the CD or provided by the artists, and in italics for those determined by the authors (with the help of professional musicians). Only up to the three most dominant keys and scales are given for each clip. The “>” symbol indicates the relative abundance of the given keys and scales in the clip.
