# Supplementary material for: The Activity of Native Vacuolar Proton-ATPase in an Oscillating Electric Field – Demystifying an Apparent Effect of Music on a Biomolecule
Source: Front Mol Biosci. 2021 Nov 3;8:772167. doi: 10.3389/fmolb.2021.772167 (PMC8595334; doi:10.3389/fmolb.2021.772167)
Supplement: Supplementary file 2 [file Table2.pdf]

Supplementary Table S2. Optical densities (*OD*) and specific ATPase activities* (*SA* in nmole ATP min^{-1} mg^{-1}) in vacuolar vesicle dispersions (all experiments)

Audio clip	+AC, -ConcA		+AC, +ConcA		-AC, -ConcA		-AC, +ConcA	
	OD	*SA*	*OD*	*SA*	*OD***	*SA***	*OD***	*SA***
0_jarret	1.219	74.388	0.494	20.916	1.157	69.815	0.57	26.521
0_jarret	1.213	73.945	0.513	22.317	1.157	69.815	0.57	26.521
0_jarret	1.194	72.544	0.551	25.120	1.157	69.815	0.57	26.521
1_deszk	1.07	63.399	0.414	15.016	1.057	62.440	0.458	18.261
1_deszk	1.084	64.431	0.41	14.721	1.057	62.440	0.458	18.261
1_deszk	1.091	64.947	0.405	14.352	1.057	62.440	0.458	18.261
2_mozart	1.15	69.299	0.498	21.211	1.157	69.815	0.57	26.521
2_mozart	1.182	71.659	0.511	22.170	1.157	69.815	0.57	26.521
2_mozart	1.157	69.815	0.524	23.129	1.157	69.815	0.57	26.521
3_bach	1.046	61.628	0.403	14.204	1.057	62.440	0.458	18.261
3_bach	1.037	60.965	0.398	13.836	1.057	62.440	0.458	18.261
3_bach	1.043	61.407	0.424	15.753	1.057	62.440	0.548	24.899
4_deszk	1.078	63.989	0.418	15.311	1.057	62.440	0.458	18.261
4_deszk	1.104	65.906	0.432	16.343	1.057	62.440	0.458	18.261
4_deszk	1.067	63.177	0.415	15.089	1.057	62.440	0.458	18.261
5_legedi	1.183	71.733	0.536	24.014	1.157	69.815	0.57	26.521
5_legedi	1.147	69.078	0.51	22.096	1.157	69.815	0.57	26.521
5_legedi	1.168	70.627	0.515	22.465	1.157	69.815	0.57	26.521
6_mozart	1.179	71.438	0.517	22.612	1.157	69.815	0.57	26.521
6_mozart	1.168	70.627	0.568	26.374	1.157	69.815	0.57	26.521
6_mozart	1.162	70.184	0.487	20.400	1.157	69.815	0.57	26.521
7_berry	1.062	62.809	0.418	15.311	1.057	62.440	0.458	18.261
7_berry	1.064	62.956	0.435	16.565	1.057	62.440	0.458	18.261
7_berry	1.07	63.399	0.403	14.204	1.057	62.440	0.458	18.261
8_boneym	1.031	60.522	0.423	15.679	1.057	62.440	0.458	18.261
8_boneym	1.054	62.219	0.415	15.089	1.057	62.440	0.458	18.261
8_boneym	1.048	61.776	0.398	13.836	1.057	62.440	0.458	18.261
9_bach	1.04	61.186	0.403	14.204	1.057	62.440	0.458	18.261
9_bach	1.022	59.858	0.43	16.196	1.057	62.440	0.458	18.261
9_bach	1.06	62.661	0.435	16.565	1.057	62.440	0.458	18.261
10_fchoir	1.175	71.143	0.573	26.743	1.157	69.815	0.57	26.521
10_fchoir	1.209	73.650	0.631	31.020	1.157	69.815	0.57	26.521
10_fchoir	1.171	70.848	0.569	26.448	1.157	69.815	0.57	26.521
11_liszt	1.166	70.479	0.516	22.539	1.157	69.815	0.57	26.521
11_liszt	1.137	68.340	0.607	29.250	1.157	69.815	0.57	26.521
11_liszt	1.148	69.151	0.569	26.448	1.157	69.815	0.57	26.521
12_liszt	1.164	70.332	0.604	29.029	1.157	69.815	0.57	26.521
12_liszt	1.145	68.930	0.578	27.111	1.157	69.815	0.57	26.521

12_liszt	1.152	69.446	0.541	24.382	1.157	69.815	0.57	26.521
13_wnoise	1.09	64.874	0.446	17.376	1.024	60.006	0.371	11.844
13_wnoise	1.078	63.989	0.404	14.278	1.024	60.006	0.371	11.844
13_wnoise	1.00	58.236	0.389	13.172	1.024	60.006	0.371	11.844
14_abba	0.961	55.359	0.342	9.705	1.024	60.006	0.371	11.844
14_abba	1.018	59.563	0.376	12.213	1.024	60.006	0.371	11.844
14_abba	0.962	55.433	0.369	11.697	1.024	60.006	0.371	11.844
15_jarre	1.16	70.037	0.562	25.931	1.157	69.815	0.57	26.521
15_jarre	1.135	68.193	0.597	28.513	1.157	69.815	0.57	26.521
15_jarre	1.099	65.537	0.605	29.103	1.157	69.815	0.57	26.521
16_pnoise	0.946	54.253	0.473	19.367	1.024	60.006	0.371	11.844
16_pnoise	0.981	56.834	0.365	11.402	1.024	60.006	0.371	11.844
16_pnoise	0.93	53.073	0.377	12.287	1.024	60.006	0.371	11.844
17_bach	0.922	52.483	0.467	18.925	1.024	60.006	0.371	11.844
17_bach	1.013	59.195	0.461	18.482	1.024	60.006	0.371	11.844
17_bach	0.984	57.056	0.42	15.458	1.024	60.006	0.371	11.844
18_deszk	1.127	67.603	0.668	33.749	1.157	69.815	0.57	26.521
18_deszk	1.162	70.184	0.685	35.003	1.157	69.815	0.57	26.521
18_deszk	1.152	69.446	0.738	38.912	1.157	69.815	0.57	26.521
19_vivaldi	0.974	56.318	0.477	19.662	1.024	60.006	0.371	11.844
19_vivaldi	0.996	57.941	0.537	24.087	1.024	60.006	0.371	11.844
19_vivaldi	0.964	55.581	0.535	23.940	1.024	60.006	0.371	11.844

*Calculated using the equation $SA = (OD - 0.21041)/(0.0045195 * 0.3 * 10)$ nmole ATP min^{-1} mg^{-1} based on the total protein content (0.3 mg) per sample, 10 min reaction time and the inorganic phosphate calibration in the same system published earlier[15].

**Identical numbers for the control (no AC filed) samples are those of the corresponding isolation batches (several samples were made from each batch).
